# The Reducing Agents in Sonochemical Reactions without Any Additives

**DOI:** 10.3390/molecules28104198

**Published:** 2023-05-19

**Authors:** Kyuichi Yasui

**Affiliations:** National Institute of Advanced Industrial Science and Technology (AIST), Nagoya 463-8560, Japan; k.yasui@aist.go.jp

**Keywords:** sonochemistry, reducing agents, an air or argon bubble, ultrasound, numerical simulations, bubble collapse, chemical reactions, water vapor, H atoms

## Abstract

It has been experimentally reported that not only oxidation reactions but also reduction reactions occur in aqueous solutions under ultrasound without any additives. According to the numerical simulations of chemical reactions inside an air or argon bubble in water without any additives under ultrasound, reducing agents produced from the bubbles are $H,{\;H}_{2},{\;\mathrm{HO}}_{2}$ (which becomes superoxide anion ($O_{2}^{-}$) in liquid water), NO, and ${\mathrm{HNO}}_{2}$ (which becomes ${\mathrm{NO}}_{2}^{-}$ in liquid water). In addition, $H_{2}O_{2}$ sometimes works as a reducing agent. As the reduction potentials of H and $H_{2}$ (in strongly alkaline solutions for $H_{2}$) are higher than those of RCHOH radicals, which are usually used to reduce metal ions, H and $H_{2}$ generated from cavitation bubbles are expected to reduce metal ions to produce metal nanoparticles (in strongly alkaline solutions for $H_{2}$ to work). It is possible that the superoxide anion ($O_{2}^{-}$) also plays some role in the sonochemical reduction of some solutes. In strongly alkaline solutions, hydrated electrons ($e^{-}\left(\mathrm{aq}\right)$) formed from H atoms in liquid water may play an important role in the sonochemical reduction of solutes because the reduction potential is extremely high. The influence of ultrasonic frequency on the amount of H atoms produced from a cavitation bubble is also discussed.

## 1. Introduction

An ultrasonic bath is widely used for cleaning glass, medical equipment, etc. [1,2]. Furthermore, an ultrasonic bath is widely used in laboratories for the dispersion of nanoparticles in various liquids [3,4,5,6]. An ultrasonic horn is also widely used in the dispersion of nanoparticles as well as in the dissolution of gels [3,4,5,6,7,8,9,10,11]. The physical and chemical effects of strong ultrasound are mostly caused by acoustic cavitation, which is the formation of gas bubbles and the violent collapse of the bubbles under ultrasound [2]. When the acoustic pressure amplitude is larger than the cavitation threshold, many tiny bubbles of a few micrometers in diameter are created [2,12,13,14,15,16]. During the rarefaction phase of ultrasound, the bubbles expand. At the compression phase of ultrasound, some of the bubbles violently collapse, which is called the Rayleigh collapse [2,17,18]. The reasons for the violent bubble collapse are as follows [2,18]. One is the inertia of the inflowing liquid toward the bubble during the bubble collapse. The other is the nearly spherical geometry of the bubble collapse, as the continuity of the liquid requires an increase in the inward liquid velocity as the distance from the bubble center decreases because the surface area decreases. The violent collapse stops when the gas density inside the bubble increases nearly to the density of liquid (the condensed phase) because the internal pressure of the bubble significantly increases [2,19]. At the final moment of the violent bubble collapse, the temperature and pressure inside the bubble increase to several thousand Kelvin and several hundred of atmospheric pressure or more because the work performed on a collapsing bubble by the surrounding liquid heats up the bubble [2,12,19,20,21,22]. In other words, it is a quasi-adiabatic collapse, where “quasi-” means that appreciable thermal conduction takes place between the heated interior of a bubble and the surrounding liquid [12]. As a result, water vapor and oxygen, if present, are thermally dissociated inside a heated bubble, and oxidants such as OH radicals, $H_{2}O_{2}$, and O radicals are formed [2,23,24,25]. In addition, faint light is emitted from the heated bubbles, which is called sonoluminescence, partly because gases inside heated bubbles are weakly ionized [2,12,17,20,26,27,28,29,30,31,32]. The oxidants diffuse into the liquid and react with solutes, which are called sonochemical reactions [2,23,33,34,35,36,37,38,39]. Sonochemical reactions in aqueous solutions are mostly the oxidation of solutes, as easily confirmed by the chemiluminescence of luminol, which is called sonochemiluminescence [2,23,40,41,42,43,44,45,46,47,48,49,50]. The oxidation of potassium iodide (Reaction (1)) is widely used as a dosimeter to calibrate sonochemical efficiency [2,51,52].

(1) $$2OH+3I^{-}\to 2OH^{-}+I_{3}^{-}$$

The chemical products from cavitation bubbles under ultrasound are mostly oxidants because H atoms, which are reducing agents, formed inside a bubble by the dissociation of water vapor hardly penetrate into the liquid phase due to the chemical reactions with $O_{2}$ and OH inside a bubble as follows [53].

(2) $$H+O_{2}\to O+OH$$

(3) $$H+O_{2}+M\to HO_{2}+M$$

(4) $$H+OH\to H_{2}+O$$

On the other hand, the sonochemical reduction of metal ions to produce metal nanoparticles has been experimentally reported [54,55,56,57,58,59,60,61,62,63,64,65,66,67,68,69,70,71,72,73]. In most of the experiments, an organic material such as alcohol ($RCH_{2}OH$, where R=H or alkyl group), ascorbic acid, formic acid, etc., was added to the aqueous solution to produce a reducing agent by the chemical reaction with OH radicals generated from cavitation bubbles as follows for alcohol [54,55,56,57,58,59,60,61,65,66,68,71,72,73].

(5) $$RCH_{2}OH+OH\to RCHOH+H_{2}O$$

where RCHOH is a reducing agent (radical) and reduces metal ions as follows [57,61,68].

(6) $$3RCHOH+AuCl_{4}^{-}\to 3RCHO+3H^{+}+Au+4Cl^{-}$$

Citric acid and EDTA also produce reducing agents by reacting with OH radicals [74,75].

In 2020, Okitsu et al. [76] reported that in the experiment of sonochemical reduction of $AuCl_{4}^{-}$ in aqueous butanol ($CH_{3}{\left(CH_{2}\right)}_{3}OH$) solution under Ar at 200 kHz, the main reducing agents were CO, $CH_{3}$, and other products of pyrolysis of butanol by acoustic cavitation. It has been known that volatile solutes such as low-molecular-weight alcohols enter cavitation bubbles and are dissociated in the heated bubbles at the violent bubble collapse [2,77]. Kamali et al. [78] suggested that zero-valent copper was formed by the thermal decomposition of copper (II) acetate monohydrate ($Cu{\left(CO_{2}CH_{3}\right)}_{2}\cdot H_{2}O$) inside heated cavitation bubbles at the violent bubble collapse. However, in their experiment [78], an ethylene glycol-ethanol solution (1:1) was used, and reducing agents might be formed by the thermal decomposition of ethylene glycol and/or ethanol. Further studies are required on this topic.

On the other hand, there are a few experimental reports that metal ions are reduced in sonochemical reactions without any additives such as organic materials, as discussed in more detail in the following section [68,69,70]. In the present review, reducing agents in sonochemical reactions without any additives are discussed based on numerical simula-tions of chemical reactions inside an air or argon bubble [24,53]. One of the aims of the present review is to stress that not only oxidation reactions but also reduction reactions occur in sonochemical reactions without any additives. Another aim is to discuss the chall-enges faced in this field and the outlook for research direction. The other aim is to provide information to enhance the reduction reactions in sonochemical reactions without adding any additives.

## 2. Sonochemical Reduction without Any Additives

In 2009, Sakai et al. [69] experimentally reported the sonochemical reduction of $AuCl_{4}^{-}$ ions in an aqueous $HAuCl_{4}$ solution in the concentration range of 0.01–0.1 mM without any additives. The solution was irradiated with ultrasound in an ultrasonic bath, which enabled it to generate 28, 200, and 950 kHz ultrasound. The liquid temperature ranged from 4 to 60 °C. Ultra-pure water used in the experiment was purged by argon gas to promote radical (H and OH) formation from water. The proposed chemical reactions in the solution under ultrasound are as follows [69].

(7) $$2Au^{III}Cl_{4}^{-}+H\to 2Au^{II}Cl_{3}^{-}+Cl+HCl$$

(8) $$2Au^{II}Cl_{3}^{-}\to Au^{III}Cl_{4}^{-}+Au^{I}Cl_{2}^{-}$$

(9) $$Au^{I}Cl_{2}^{-}+H\to Au^{0}+HCl+Cl^{-}$$

(10) $$nAu^{0}\to {\left(Au^{0}\right)}_{n}$$

(11) $$\left(2Au^{I},Au_{n}\right)\to \left(Au^{II},Au_{n+1}\right)$$

(12) $$Au^{III}Cl_{4}^{-}+2Au^{0}+2Cl^{-}↔3Au^{I}Cl_{2}^{-}$$

In the experiment of Sakai et al. [69], the reduction of $AuCl_{4}^{-}$ was monitored by the decrease of the $AuCl_{4}^{-}$ concentration ($\left[Au^{III}\right])$ in solutions with an inductively coupled plasma spectrometer (ICPS) (ICPS-7500, SHIMADZU, Kyoto, Japan). The formation of gold nanoparticles was confirmed by observing the absorption spectra at ~530 nm originating from the surface plasmon resonance of the gold nanoparticles using a UV-visible spectrometer. The size and shape of the produced gold nanoparticles were observed by transmission electron microscopy (TEM) (H-7650, Hitachi High Technologies Co., Tokyo, Japan).

**Figure 1:**
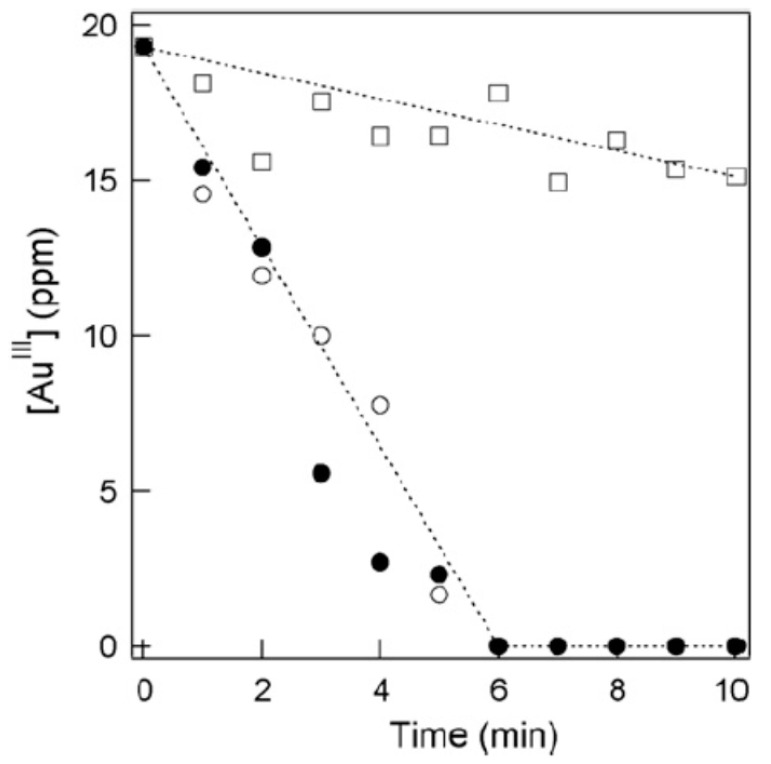
The concentration of ${\mathrm{AuCl}}_{4}^{-}$ ($\left[{\mathrm{Au}}^{\mathrm{III}}\right]$) as a function of sonication time, using different frequencies of ultrasound: 28 kHz (☐), 200 kHz (○), and 950 kHz (●) at 25 °C. The initial concentration of ${\mathrm{AuCl}}_{4}^{-}$ was 0.1 mM. Reprinted with permission from Ref. [69]. Copyright 2009, Elsevier.

Experimental evidence for the sonochemical reduction of $AuCl_{4}^{-}$ without any additive is the decrease of $AuCl_{4}^{-}$ concentration with time measured with ICPS, as shown in Figure 1 [69]. The concentration of $AuCl_{4}^{-}$ ions in the solution decreased with sonication time and became zero at ~6 min in the case of 200 and 950 kHz sonication (Figure 1). In the case of 28 kHz sonication, the decrease in $AuCl_{4}^{-}$ concentration was not significant. They indicate that higher-frequency ultrasound is more effective at reducing $AuCl_{4}^{-}$ than lower-frequency ultrasound. In the case of 200 and 950 kHz sonication, an absorption band centered at ~530 nm originated from the surface plasmon resonance of the gold nanoparticles was observed using a UV-visible spectrometer [69]. In the case of 28 kHz, there was no noticeable peak in the absorption spectrum. The facts also indicate that appreciable amounts of gold nanoparticles were produced for the cases of 200 and 950 kHz sonication, while much less gold nanoparticles were produced for the case of 28 kHz sonication. 

**Figure 2:**
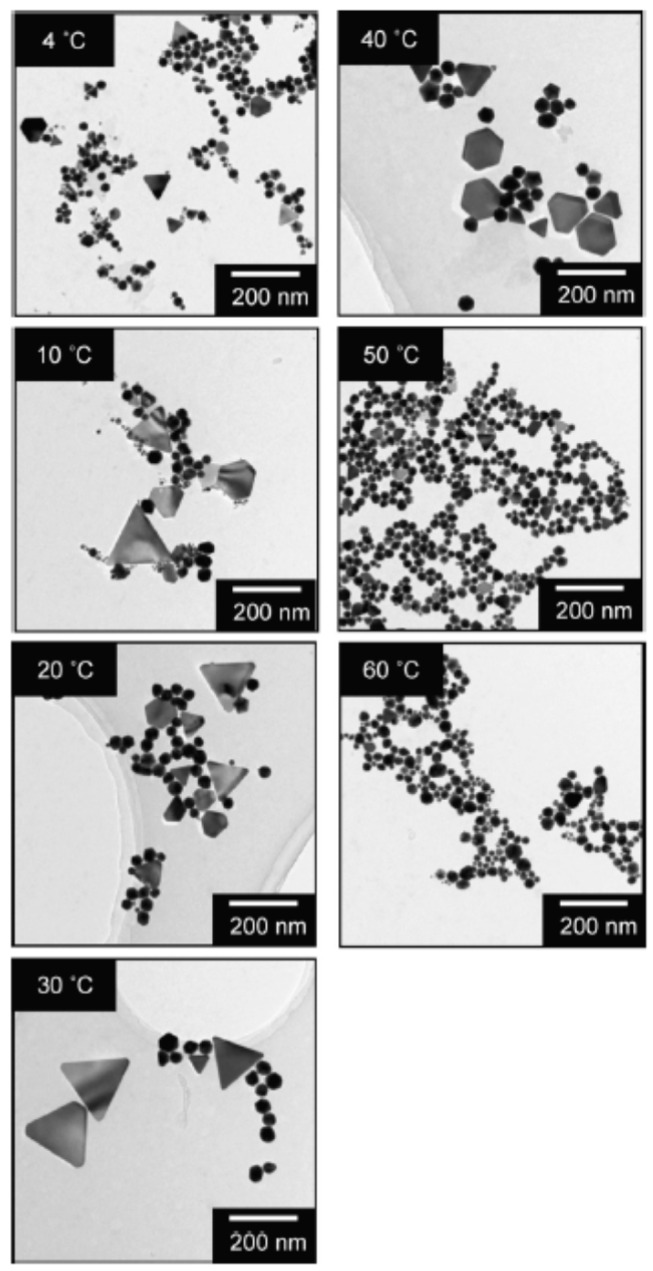
TEM images of gold nanoparticles formed from aqueous 0.1 mM ${\mathrm{AuCl}}_{4}^{-}$ solutions by 950 kHz sonication for 8 min at 4, 10, 20, 30, 40, 50, and 60 °C. Reprinted with permission from Ref. [69]. Copyright 2009, Elsevier.

According to the TEM observation of the produced gold nanoparticles formed from aqueous 0.1 mM $AuCl_{4}^{-}$ solutions by 950 kHz sonication for 8 min, there were spherical nanoparticles with a diameter of 20–60 nm and triangular and/or hexagonal plates with a size of 20–200 nm, as shown in Figure 2 [69]. As seen in Figure 2, the size and shape of the produced gold nanoparticles depend on the liquid temperature during the sonication. For the temperature range of 4–40 °C, triangular and hexagonal plates were formed along with the spherical nanoparticles. On the other hand, for the cases of 50 and 60 °C, only spherical gold nanoparticles were formed, as shown in Figure 2 [69]. Sakai et al. [69] suggested that under relatively high liquid temperatures, dissolution of triangular and hexagonal plates would be accelerated because, 30 days after the preparation of gold nanoparticles at 25 °C, triangular and hexagonal plates disappeared. With regard to the mechanism for the formation of triangular and hexagonal plates, Sakai et al. [69,70] suggested that $Cl^{-}$ ions are adsorbed on a certain crystal facet and reduction of $AuCl_{4}^{-}$ ions occurs on other crystal facets. Indeed, the formation of triangular and/or hexagonal plates was promoted with the addition of NaCl, and the size of the plate increased as the NaCl concentration increased [69]. The larger size of triangular and hexagonal plates compared to spherical nanoparticles may be due to the accelerated reduction of $AuCl_{4}^{-}$ ions on the crystal facets of the plates. Further studies are required on this topic. 

**Figure 3:**
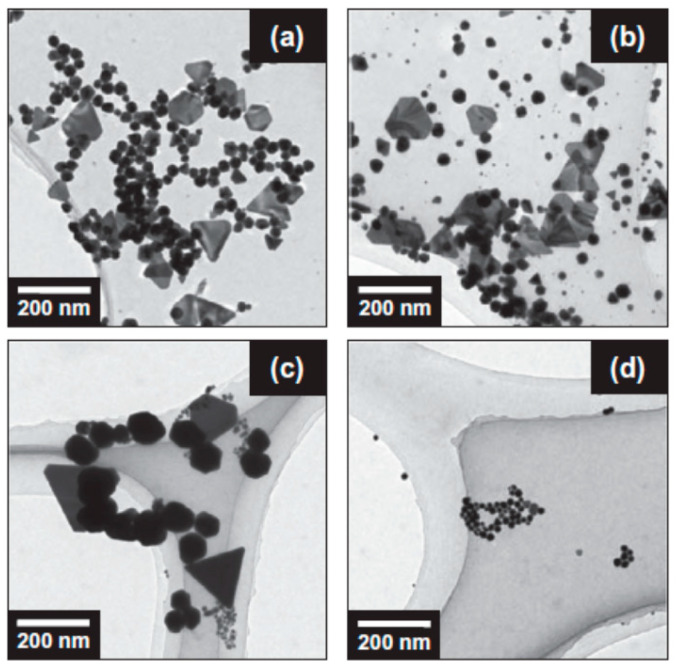
TEM images of gold nanoparticles formed from (**a**) Ar-, (**b**) $N_{2}$-, (**c**) $O_{2}$-, and (**d**) $H_{2}$-purged aqueous 0.1 mM $AuCl_{4}^{-}$ solutions by 950 kHz sonication for 8 min at 25 °C. The solutions were purged with gas for 30 min. Reprinted with permission from Ref. [70]. Copyright 2014, Elsevier.

**Figure 4:**
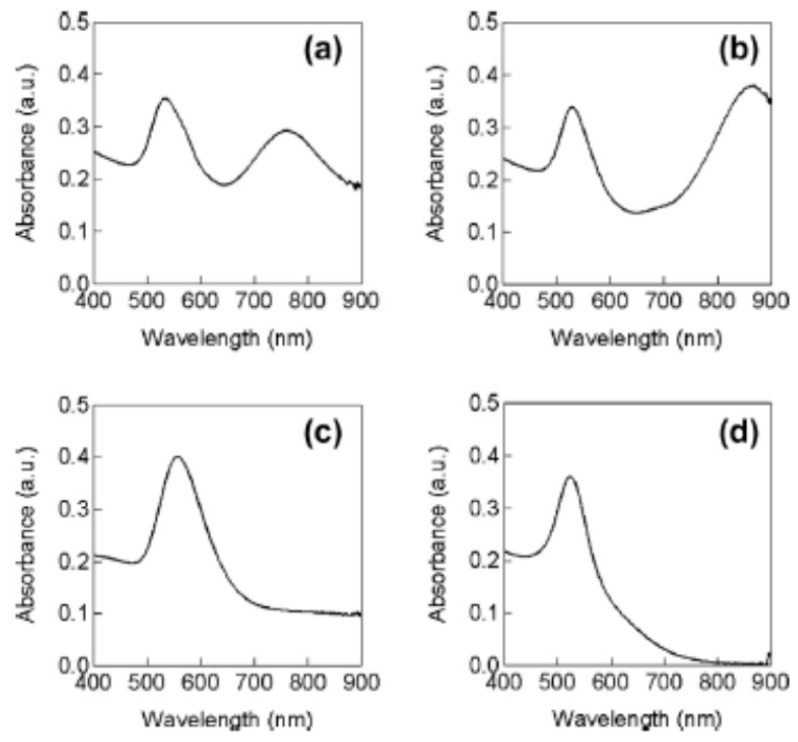
Absorption spectra originating from surface plasmon resonance of gold nanoparticles formed from (**a**) Ar-, (**b**) $N_{2}$-, (**c**) $O_{2}$-, and (**d**) $H_{2}$-purged aqueous 0.1 mM $AuCl_{4}^{-}$ solutions by 950 kHz sonication for 8 min at 25 °C. Reprinted with permission from Ref. [70]. Copyright 2014, Elsevier.

**Figure 5:**
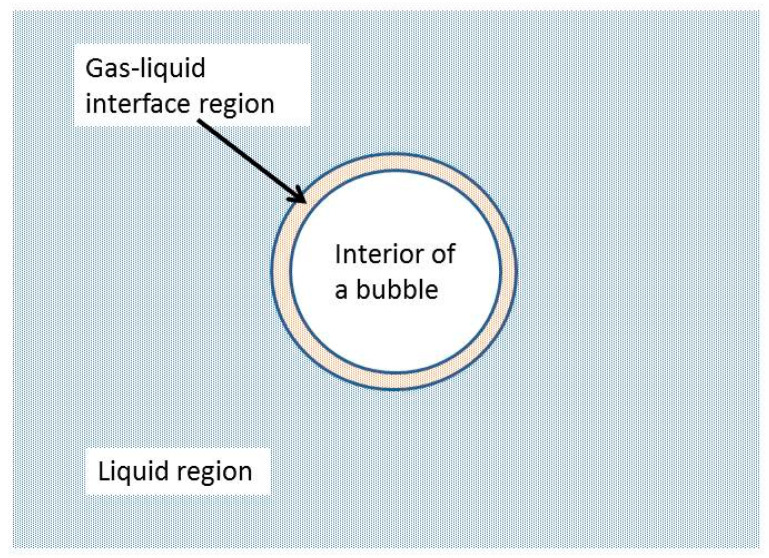
Three regions for a cavitation bubble. Reprinted with permission from Ref. [82]. Copyright 2016, Springer Nature.

In 2014, Sakai et al. [70] experimentally reported the influence of gas species on the sonochemical reduction of $AuCl_{4}^{-}$ without any additives (Figure 3 and Figure 4). The gas species studied were $Ar,N_{2},O_{2},$ and $H_{2}$. Aqueous 0.1 mM $AuCl_{4}^{-}$ solutions were purged with each gas for 30 min before sonication [70]. The solutions were sonicated in an ultrasonic bath at 950 kHz for 8 min at 25 °C [70]. For an Ar-purged solution, spherical and plate-like gold nanoparticles were produced (Figure 3a). Indeed, bimodal absorption bands centered at ~520 and ~720 nm originated from the surface plasmon resonance of the spherical and plate-like gold nanoparticles, respectively, were observed (Figure 4a). For a $N_{2}$-purged solution, the gold nanoparticles produced were similar to those for the Ar-purged solution (Figure 3b). However, the size of plate-like gold nanoparticles produced from the $N_{2}$-purged solution seems to be larger than those from the Ar-purged solution because the absorption band was shifted to a longer wavelength (Figure 4b) [70,79]. For an $O_{2}$-purged solution, larger gold nanoparticles were produced compared to those for the Ar-purged and $N_{2}$-purged solutions (Figure 3c and Figure 4c) [70]. On the other hand, for a $H_{2}$-purged solution, spherical gold nanoparticles of ~20 nm were selectively produced (Figure 3d and Figure 4d) [70]. It should be noted that gas purging alone without sonication did not reduce $AuCl_{4}^{-}$ in aqueous solutions [70]. Sakai et al. [70] suggested that the formation of smaller spherical gold nanoparticles in the $H_{2}$-purged solution is due to the increase in the number of nanoparticle nucleation sites caused by the increase in the amount of the reducing agent (H atoms) by the following reaction.

(13) $$OH+H_{2}\to H_{2}O+H$$

where OH radicals are produced by the thermal dissociation of water vapor in the heated hydrogen bubbles at the violent bubble collapse [80,81]. Although Sakai et al. [70] did not comment on the site where the reaction (13) occurs, it seems that the reaction (13) occurs at the gas-liquid interface region shown in Figure 5 [82]. Sakai et al. [70] discussed that the reducing agent (H atoms) produced inside oxygen bubbles from water vapor at the violent bubble collapse is consumed by the following reaction in an $O_{2}$-purged solution.

(14) $$H+O_{2}\to HO_{2}$$

Indeed, according to the numerical simulations of chemical reactions inside an oxygen ($O_{2}$) bubble in water irradiated by 22 kHz and 1.68 bar ultrasound [83], the main chemical products are H and $HO_{2}$, along with $H_{2},H_{2}O_{2}$, and OH radicals. It seems that H atoms are consumed in the gas-liquid interface region by the reaction (14). Accordingly, the number of nanoparticle nucleation sites is decreased in an $O_{2}$-purged solution, and the particle size is increased due to the smaller number of particles generated. Although Sakai et al. [70] also discussed the reason for the similarity in gold nanoparticle production in Ar-purged and $N_{2}$-purged solutions, it seems to be unclear at present. 

**Figure 6:**
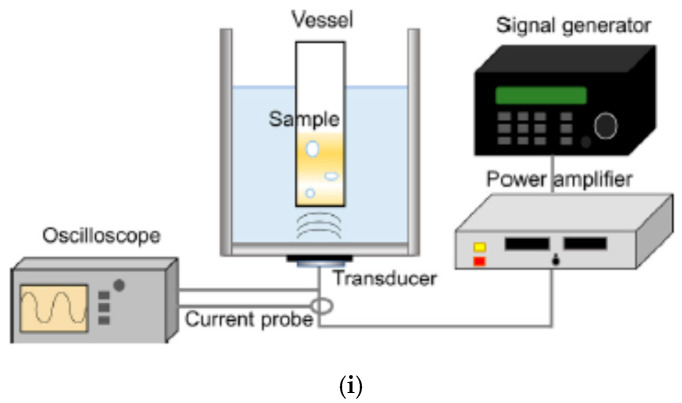

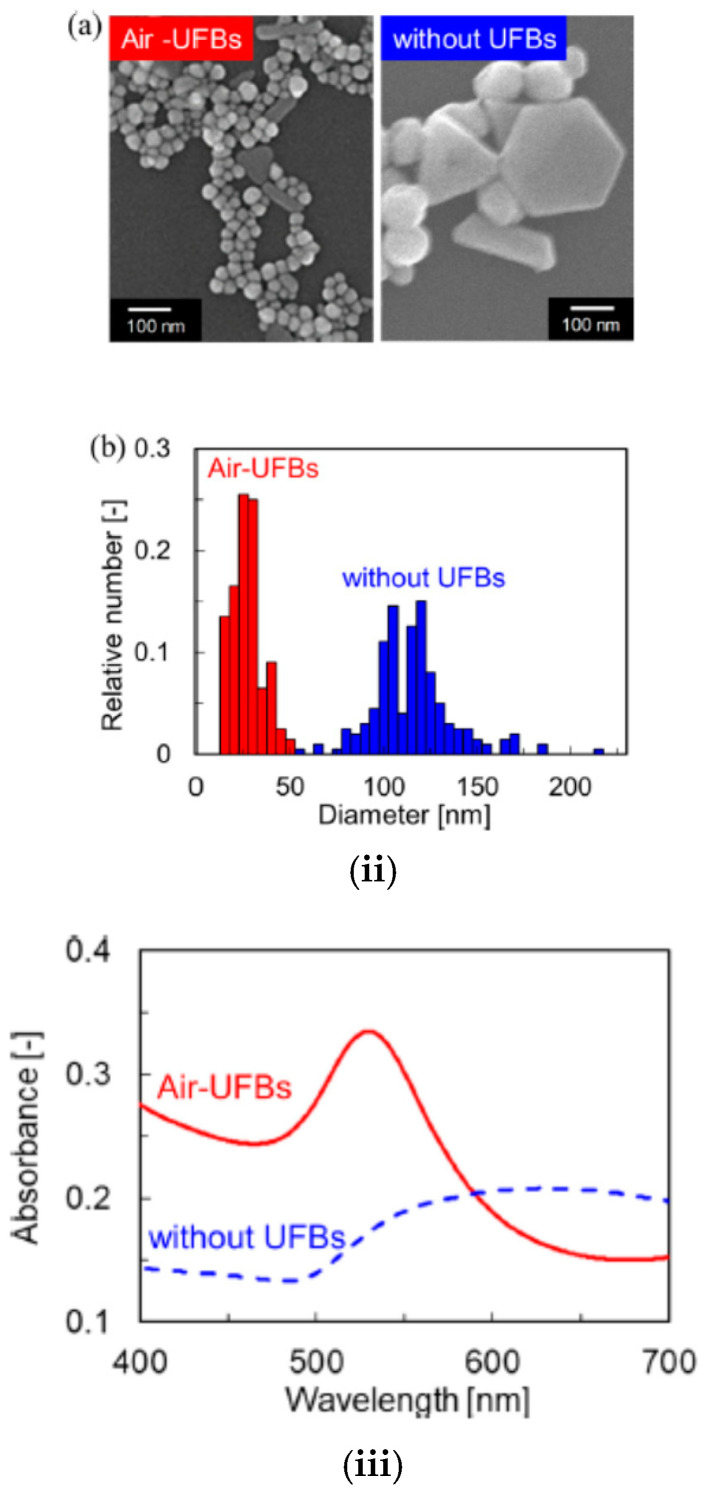
Sonochemical reduction of aqueous 0.1 mM ${\mathrm{AuCl}}_{4}^{-}$ solutions by 495 kHz sonication for 10 min at 10 °C to produce gold nanoparticles with and without ultrafine bubbles. (**i**) Experimental apparatus. (**ii**) (**a**) SEM images of gold nanoparticles. (**b**) The size distribution of spherical gold nanoparticles. (**iii**) Absorption spectra. Reprinted with permission from Ref. [84]. Copyright 2020, Elsevier.

In 2020, Yasuda et al. [84] experimentally reported the sonochemical reduction of $AuCl_{4}^{-}$ without any additives such as organic materials with and without ultrafine bubbles (UFBs, which are also called bulk nanobubbles). In the experiment [84], UFBs were generated by a pressurized dissolution method using ultrapure water and a commercially available UFB generator. Air, argon, oxygen, and nitrogen were used as gases to generate UFBs [84]. During the generation of UFBs, the liquid becomes milky due to the generation of a huge number of microbubbles, mostly using hydrodynamic cavitation [85,86]. After stopping cavitation, the liquid returns to being transparent as most of the microbubbles disappear at the liquid surface due to buoyancy. In the transparent liquid water, UFBs are present, which can be confirmed by particle tracking analysis (PTA) to estimate the number concentration and size distribution of nano and submicron particles suspended in a liquid by observing the Brownian motion of the particles with a video camera [85,87]. The size distribution of micro and ultrafine bubbles in the initial milky water is continuous, and only ultrafine bubbles of about 100 nm in diameter remain in the transparent liquid [87,88]. In the experiment of Yasuda et al. [84], the modal diameters of UFBs for all the gases were approximately 120 nm. The concentration of air-UFBs before ultrasonic irradiation was $5\times 10^{9}$ mL^−1^ [84]. It should be noted that UFBs are generally very stable, and the lifetime could be more than 200 days with a slight change in size distribution [88,89]. With regard to the mechanism of stability of a UFB against dissolution, the dynamic equilibrium model that a UFB is partly covered with hydrophobic materials (impurities) seems promising because there is evidence in the TEM images of UFBs partly covered with hydrophobic materials [90,91,92,93,94,95,96]. In the experiment of Yasuda et al. [84], water containing UFBs was used as solvent for aqueous 0.1 mM $AuCl_{4}^{-}$ solutions. The solution was irradiated with 495 kHz ultrasound using the apparatus shown in Figure 6i [84]. As is usually the case in experiments in sonochemistry [2,97], a sinusoidal electric signal is amplified with a power amplifier and supplied to an ultrasonic transducer, which is attached to the bottom of an ultrasonic bath. A vessel that contained the solution was immersed in the ultrasonic bath filled with water, as shown in Figure 6i. The comparison between sonochemically produced gold nanoparticles with and without air-UFBs is shown in Figure 6ii,iii. Without UFBs, the produced gold nanoparticles were not only spherical nanoparticles but also plate-like particles (Figure 6ii(a)). The mean diameter of the particles without UFBs was 119 nm [84]. On the other hand, with air-UFBs, mostly spherical gold nanoparticles were produced. Furthermore, the gold nanoparticles produced were much smaller than those without UFBs. The mean diameter of gold nanoparticles with air-UFBs was 22 nm [84]. This is confirmed by the absorption spectra because the absorption peak became sharper and was shifted to a shorter wavelength with air-UFBs compared to that without UFBs (Figure 6iii) [79,84]. For all the gases for UFBs studied by Yasuda et al. [84], the produced gold nanoparticles were mostly spherical nanoparticles, in contrast to the case without UFBs. The mean diameter of the produced gold nanoparticles depended on the gas species: 49 nm for Ar-UFBs, 43 nm for $O_{2}$-UFBs, 73 nm for $N_{2}$-UFBs, and 22 nm for air-UFBs [84]. Yasuda et al. [84] suggested that acoustic cavitation could be enhanced in the presence of UFBs, and accordingly, the concentration of reducing species could be increased. It may result in an increase in nucleation sites in nanoparticles and a decrease in particle size [84]. On the other hand, according to the dynamic equilibrium model of UFBs [90], a UFB is stabilized against dissolution by being partly covered with hydrophobic impurities such as oils, carbon particles, etc. The hydrophobic impurities could be produced from a UFB generator, such as abrasion powder from the mechanical seal or lubrication oil in a water pump, even if ultrapure water is used [98,99]. It suggests that sonication of the hydrophobic impurities may result in the production of reducing agents such as $CO,CH_{3},$ etc. It may also cause an increase in the nucleation sites of gold nanoparticles. With regard to the smallest nanoparticle size with air-UFBs, sonochemically produced NO and/or $NO_{2}^{-}$ might play some role [24,53]. Further studies are required on these topics. 

**Figure 7:**
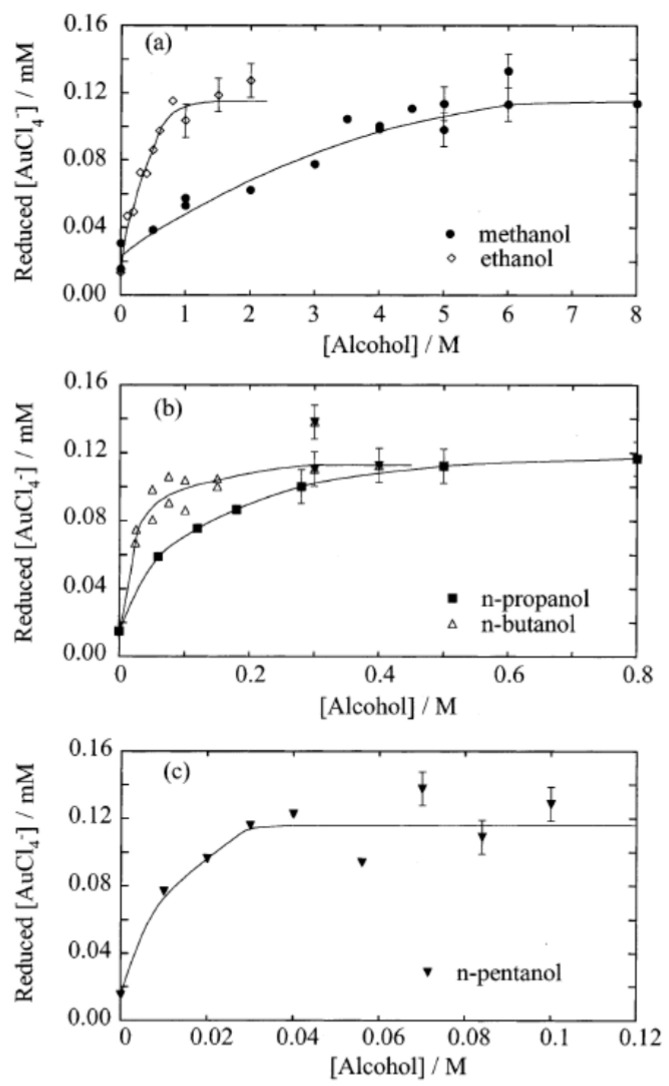
The amount of ${\mathrm{AuCl}}_{4}^{-}$ reduced by sonication for 2.5 min as a function of the alcohol concentration for (**a**) methanol and ethanol, (**b**) 1-propanol and 1-butanol, and (**c**) 1-pentanol. The initial concentration of ${\mathrm{AuCl}}_{4}^{-}$ was 0.2 mM. The pH of the solutions before and after sonication was between 3.1 and 3.5. The liquid temperature was maintained at 21 ± 3 °C. Reprinted with permission from Ref. [68]. Copyright 2002, the American Chemical Society.

In 2002, Caruso et al. [68] recognized that sonochemical reduction of $AuCl_{4}^{-}$ occurred in 0.2 mM aqueous $AuCl_{4}^{-}$ solutions without any additives at 20 °C purged with argon for 15 min before sonication. In the experiment of Caruso et al. [68], an ultrasonic horn was used for sonication at 20 kHz for 2.5 min. Gold nanoparticles were produced without any additives such as alcohol, although not as much as when alcohol was added to the solution [68]. It can be seen from Figure 7 that the amount of reduced $AuCl_{4}^{-}$ was non-zero even in the absence of alcohols (at an alcohol concentration of zero) [68]. 

## 3. Results of Numerical Simulations and Discussion

**Figure 8:**
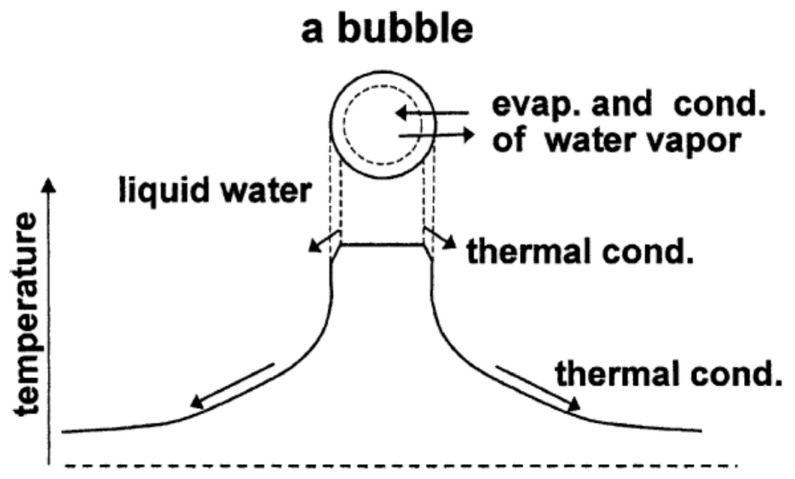
The model of bubble dynamics. The abscissa axis is spatial position. Reprinted with permission from Ref. [116]. Copyright 2004, Elsevier.

There are various theoretical models of bubble dynamics for numerical simulations of chemical reactions inside a cavitation bubble [2,16,19,24,53,77,80,100,101,102,103,104,105,106,107,108,109,110,111,112,113,114,115,116,117]. The first paper on numerical simulations of sonochemical reactions was probably by Kamath, Prosperetti, and Egolfopoulos in 1993 [100]. The model used in the numerical simulations discussed in the present paper is schematically shown in Figure 8 [116]. Temperature and pressure are assumed to be spatially uniform inside a bubble except at the thermal boundary layer near the bubble wall [2,16,19,24,53,77,80,111,112,113,114,115,116,117]. Non-equilibrium evaporation and condensation of water vapor take place at the bubble wall [115]. Thermal conduction takes place both inside and outside a bubble [19]. The temporal variation of liquid temperature at the bubble wall is numerically calculated using a rather simple model [112]. Non-equilibrium chemical reactions are taken into account by numerically calculating chemical reaction rates using Arrhenius-type rate constants [19,114]. For an air bubble, rates of 93 chemical reactions and their backward reactions are numerically calculated as a function of time involving $N_{2},O_{2},H_{2}O,OH,H,O,HO_{2},H_{2}O_{2},O_{3},N,HNO_{2},HNO$,$HNO_{3}$, NO, $NO_{2}$, and $N_{2}O$ [114,118,119,120]. Kalmar et al. [105] pointed out that the results of numerical simulations strongly depend on the chemical kinetics model employed in the simulations. In the present model, the ionization of gases and vapor inside a heated bubble is taken into account, considering the ionization-potential lowering due to high density inside a bubble at the end of the violent bubble collapse [16,113]. The model has been validated through the study of single-bubble sonochemistry [24]. The single-bubble system is as follows. A single stably pulsating bubble is trapped near the pressure antinode of a standing ultrasonic wave due to the acoustic radiation force called the primary Bjerknes force in degassed water [17,32,121,122]. If the liquid water is not sufficiently degassed, many cavitation bubbles appear under the irradiation of strong ultrasound, and a single-bubble system cannot be obtained. Single-bubble sonoluminescence (SBSL) is the light emission phenomenon from the single-bubble system [2,17,27,32,111,122] and was a popular topic in scientific research soon after the report by Barber and Putterman in 1991 [123] on the extremely short pulse-width of SBSL. In 2002, Didenko and Suslick [124] experimentally reported the production rate of OH radicals from a SBSL bubble, which is called single-bubble sonochemistry.

**Figure 9:**
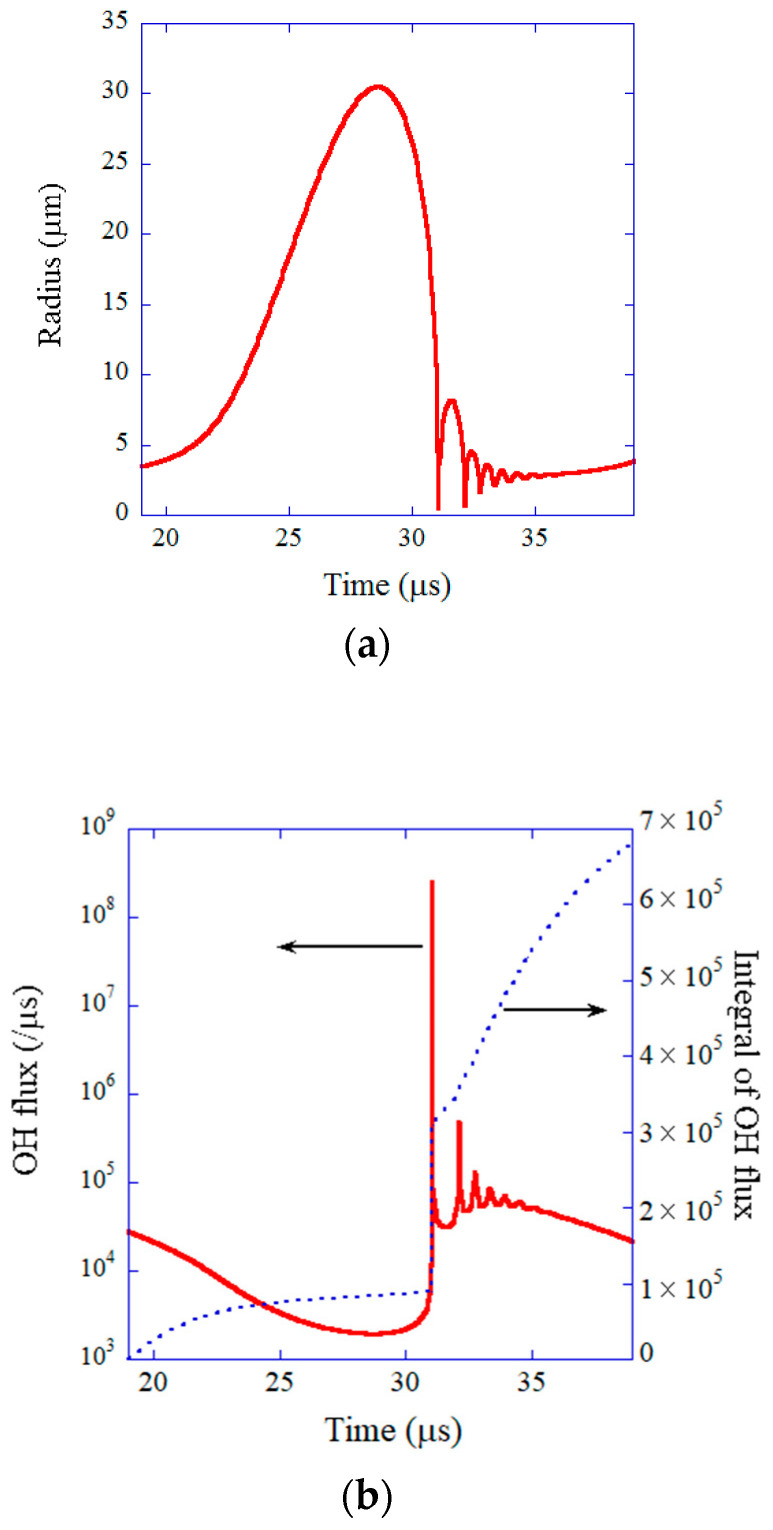
The results of the numerical simulations under the experimental condition of single-bubble sonochemistry as a function of time for one acoustic cycle under ultrasound of 52 kHz and 1.52 bar in water at 3 °C. (**a**) The bubble radius. (**b**) The dissolution rate of OH radicals into the liquid water from the interior of the bubble (red solid line) and its time integral (blue dotted line). Reprinted with permission from Ref. [24]. Copyright 2005, AIP Publishing.

**Figure 10:**
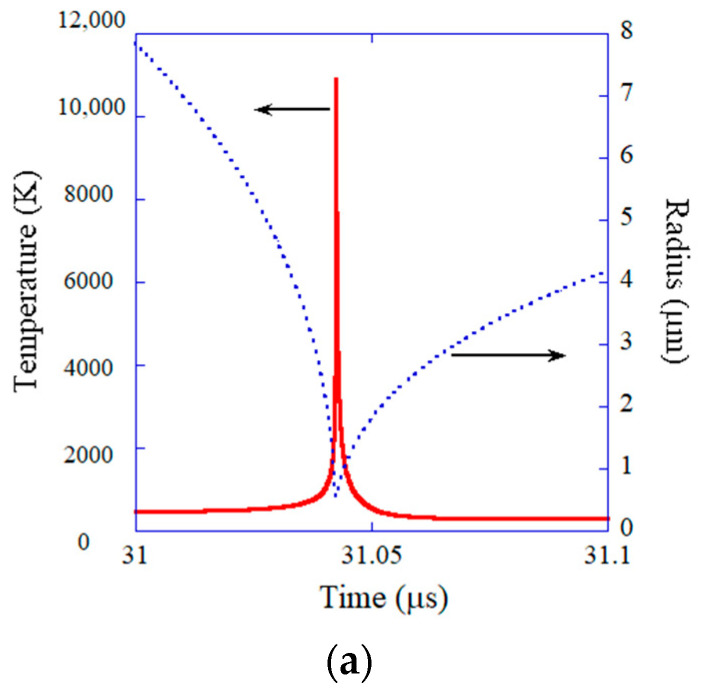

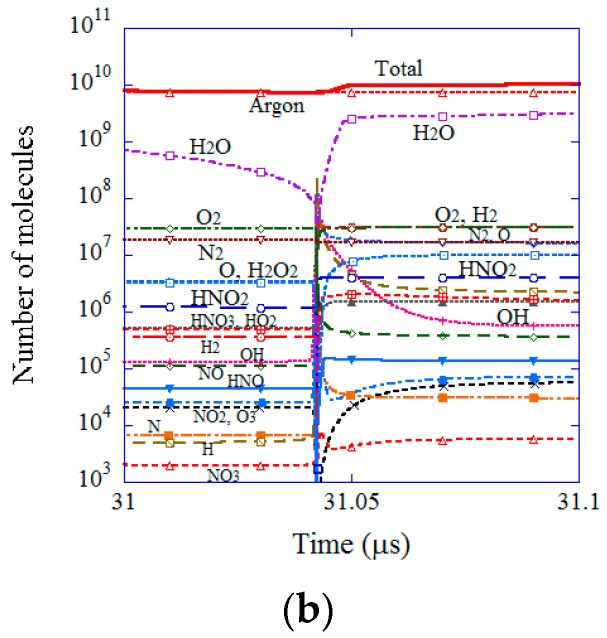
The results of the numerical simulations for a SBSL bubble (mostly an argon bubble) as a function of time at around the end of the violent bubble collapse under ultrasound of 52 kHz and 1.52 bar. (**a**) The bubble radius and the temperature inside the bubble. (**b**) The number of molecules inside the bubble. Reprinted with permission from Ref. [24]. Copyright 2005, AIP Publishing.

The results of numerical simulations under the condition of single-bubble sonochemistry are shown in Figure 9 and Figure 10 [24]. During the rarefaction phase of ultrasound, a bubble expands to a maximum radius of 30.5 μm (Figure 9a) [24,124]. At the compression phase of ultrasound, a bubble violently collapses, which is the Rayleigh collapse. At the end of the violent collapse, the temperature and pressure inside a bubble increase to 10,900 K and $7.9\times 10^{9}$ Pa, respectively (Figure 10a for temperature) [24]. As a result, almost all water vapor molecules inside a bubble are dissociated at the end of the violent bubble collapse, and an appreciable amount of OH radicals are produced inside the bubble (Figure 10b). It should be noted that the main content of a SBSL bubble is argon because nitrogen and oxygen chemically react inside a heated air bubble and change to $HNO_{x}$ and $NO_{x}$, which gradually dissolve into the surrounding liquid water [17,125]. After about one hundred acoustic cycles, which corresponds to one hundred violent collapses, only chemically inactive argon remains inside a bubble, as 1% of the air in the molar fraction is argon [126]. This argon rectification hypothesis has been validated both theoretically and experimentally [17]. The chemical species present inside a SBSL bubble just before the end of the violent collapse in Figure 10b were produced at the previous violent collapse [24]. The OH flux from the SBSL bubble and its time integral are shown in Figure 9b as a function of time for one acoustic cycle [24]. About 1/3 of the total amount of OH radicals that diffuse into the surrounding liquid water in one acoustic cycle diffuse out of a bubble at around the end of the violent collapse. The other 2/3 of OH radicals diffuse into the surrounding liquid during bubble expansion and the bouncing motion shown in Figure 9a. The total amount of OH radicals that diffuse into the liquid is $6.6\times 10^{5}$ in number of molecules according to the present numerical simulation, which almost agrees with the experimental data of $8.2\times 10^{5}$ by Didenko and Suslick [124]. It means that the present model has been validated by comparison with the experimental data. It should be noted that an ODE (ordinary differential equations) model like the present model needs to be validated through comparison with experimental data or the results of first-principle calculations because an ODE model is not fully based on the first principles [127].

The amounts of chemical products that dissolve into the surrounding liquid from a SBSL bubble (mostly an argon bubble) in one acoustic cycle are shown in Table 1, according to the results of the numerical simulation [24]. In order to discuss reducing agents produced from a SBSL bubble, the reduction potentials of reducing agents are listed in Table 2 [128,129,130]. Among the reducing agents produced from a SBSL bubble in Table 1, the reduction potential of the H atom is extremely high, as listed in Table 2. In other words, the main reducing agent produced by a SBSL bubble is the H atom. For an air bubble, the amount of H atoms produced from a bubble is much smaller than that from an argon bubble (a SBSL bubble), as listed in Table 3. Nevertheless, as the reduction potential of H atoms is extremely high compared to other reducing agents, the main reducing agent produced from an air bubble would also be H atoms. Experimentally, H atoms produced from argon-saturated aqueous solutions irradiated with ultrasound (which are multi-bubble systems) have been detected by spin trapping and electron spin resonance along with OH radicals [131,132,133].

For a SBSL bubble (mostly an argon bubble), the main chemical product produced from a bubble is $H_{2}$ as shown in Table 1 [24]. Indeed, $H_{2}$ produced from cavitation bubbles in water under argon irradiated with 300 kHz and 12 W of ultrasound (which was a multi-bubble system) was experimentally detected by using a mass spectrometer, and the rate of $H_{2}$ formation was 10 μM min^−1^ [134]. There are also other experimental reports that $H_{2}$ produced from cavitation bubbles in water in which $O_{2}$, air, $N_{2}$, or argon were dissolved and irradiated with ultrasound was detected [23,70,135,136,137]. There have also been some numerical studies on the hydrogen production from cavitation bubbles in recent years [138,139,140,141]. In the experiment of Sakai et al. [70], $H_{2}$-gas purging alone did not reduce $AuCl_{4}^{-}$ in aqueous solutions. Thus, the reducing power of $H_{2}$ is insufficient for the reduction of $AuCl_{4}^{-}$ as the reduction potential of $H_{2}$ in acidic solutions, which was the case in the experiments of Sakai et al. [69,70], is only 0.00 V (the standard condition) (Table 2). On the other hand, in strongly alkaline solutions, the reduction potential of $H_{2}$ could be larger than that of $CH_{2}OH$ and $CH_{3}CHOH$, which easily reduce $AuCl_{4}^{-}$ (Table 2). It suggests that $H_{2}$ produced from cavitation bubbles could work as a reducing agent in strongly alkaline solutions. $H_{2}$ is also produced from an air bubble, as listed in Table 3. 

For an air bubble, the main chemical product is $HNO_{2}$ under the conditions listed in Table 3 [24]. Indeed, $HNO_{2}$ produced from air bubbles in water at 25 °C irradiated with 447 kHz and 50 W ultrasound (which was a multi-bubble system) was detected by a diazotization method, and the rate of $HNO_{2}$ formation was 22 μM min^−1^ [142]. There have also been other experimental reports that $NO_{2}^{-}$, which is mainly formed by $HNO_{2}\to H^{+}+NO_{2}^{-}$ (pK=3.3 [143]), produced from cavitation bubbles in water irradiated with ultrasound, is detected [23,124,136,144,145]. Although the reduction potential of $HNO_{2}$ in acidic solutions is rather low, as listed in Table 2, that of $NO_{2}^{-}$ in strongly alkaline solutions is comparable to that of $H_{2}$ in acidic solutions. Further studies are required to determine whether $HNO_{2}$ and $NO_{2}^{-}$ play some role in the sonochemical reduction of solutes. It should be noted that $HNO_{2}$ and $NO_{2}^{-}$ not only work as reducing agents but also as oxidants, as listed in Table 4 [129,146].

Both for an argon bubble (a SBSL bubble) and an air bubble, one of the main chemical products is $H_{2}O_{2}$, as listed in Table 1 and Table 3 [24]. Indeed, $H_{2}O_{2}$ produced from air bubbles in water at 25 °C irradiated with 447 kHz and 50 W ultrasound was detected by the oxidation reaction of potassium iodide (KI), and the rate of $H_{2}O_{2}$ formation was 21 μM min^−1^ [142]. A similar rate of $H_{2}O_{2}$ formation from argon bubbles was also experimentally reported [23]. Although $H_{2}O_{2}$ plays an important role in the sonochemical oxidation of solutes [23,51,52], Okitsu et al. [147] experimentally reported that $H_{2}O_{2}$ sometimes works as a reducing agent, such as in the following reaction.

(15) $$2MnO_{4}^{-}+3H_{2}O_{2}\to 2MnO_{2}+3O_{2}+2OH^{-}+2H_{2}O$$

The chemical reaction (15) was confirmed to occur by the addition of $H_{2}O_{2}$ to a 0.1 mM $KMnO_{4}$ aqueous solution without ultrasonic irradiation [147]. As the reduction potential of $H_{2}O_{2}$ is the lowest among the reducing agents listed in Table 2 except NO in acidic solutions, almost all the reducing agents produced from cavitation bubbles, such as $H_{2},HO_{2}$ (which becomes superoxide anion ($O_{2}^{-}$) in liquid water), $HNO_{2}$ (which becomes $NO_{2}^{-}$ in liquid water), and NO (in strongly alkaline solutions), could possibly play some role in the sonochemical reduction of some solutes. It should be noted, however, that NO in acidic solutions does not reduce $AuCl_{4}^{-}$ because the reduction potential is even lower than $\left(-1\right)$ times the oxidation potential of $AuCl_{4}^{-}$, as listed in Table 2 and Table 4 [128,129,130,146]. 

The hydroperoxyl radical ($HO_{2}$) becomes a superoxide anion ($O_{2}^{-}$) in liquid water as follows [148].

(16) $$HO_{2}↔O_{2}^{-}+H^{+}\;\;\;pK=4.8$$

Kondo et al. [149] experimentally reported that $HO_{2}$ was detected in oxygen-saturated aqueous solutions irradiated by 50 kHz ultrasound and that there was evidence of $O_{2}^{-}$ formation. There have been a few other experimental reports on the formation of superoxide anion ($O_{2}^{-}$) in liquid water irradiated by ultrasound [150,151]. However, the role of the superoxide anion ($O_{2}^{-}$) in the sonochemical reduction of solutes is still unclear, and further studies are required on this topic. As the reduction potential of $O_{2}^{-}$ is higher than that of CO, which has been reported to reduce metal ions [76] (Table 2), it is possible that the superoxide anion ($O_{2}^{-}$) plays some role in the sonochemical reduction of some solutes. 

Finally, the role of hydrated electrons ($e^{-}\left(aq\right)$) produced from cavitation bubbles is discussed. In strongly alkaline solutions, hydrated electrons ($e^{-}\left(aq\right)$) are formed from H atoms as follows [23,81,152].

(17) $$H+OH^{-}↔H_{2}O+e^{-}\left(aq\right)\;\;\;\;\;pK=9.8$$

As the reduction potential of hydrated electrons is very high (Table 2), the reduction of thallium ions was experimentally reported in strongly alkaline solutions in which an argon-hydrogen mixture was dissolved and irradiated with 1 MHz ultrasound as follows [23,81].

(18) $$Tl^{+}+e^{-}\left(aq\right)\to Tl^{0}$$

As a result, colloidal thallium was formed. Thallium ions were not reduced in neutral or weakly alkaline solutions [23,81]. On the other hand, hydrated electrons ($e^{-}\left(aq\right)$) are possibly produced from plasma formed inside heated cavitation bubbles as there are free electrons in the plasma [2,12,16,17,20,24,26,27,28,29,30,31,32,103,109,113,153,154,155]. At present, however, hydrated electrons ($e^{-}\left(aq\right)$) have been experimentally detected only from a moving SBSL bubble in neutral or acidic solutions [29,156]. In a multi-bubble system at neutral pH, no detectable yield of hydrated electrons in argon-saturated aqueous solutions irradiated with 50 kHz ultrasound at 20 °C was found experimentally [157]. Further studies are required on the role of hydrated electrons in the sonochemical reduction of solutes. 

**Figure 11:**
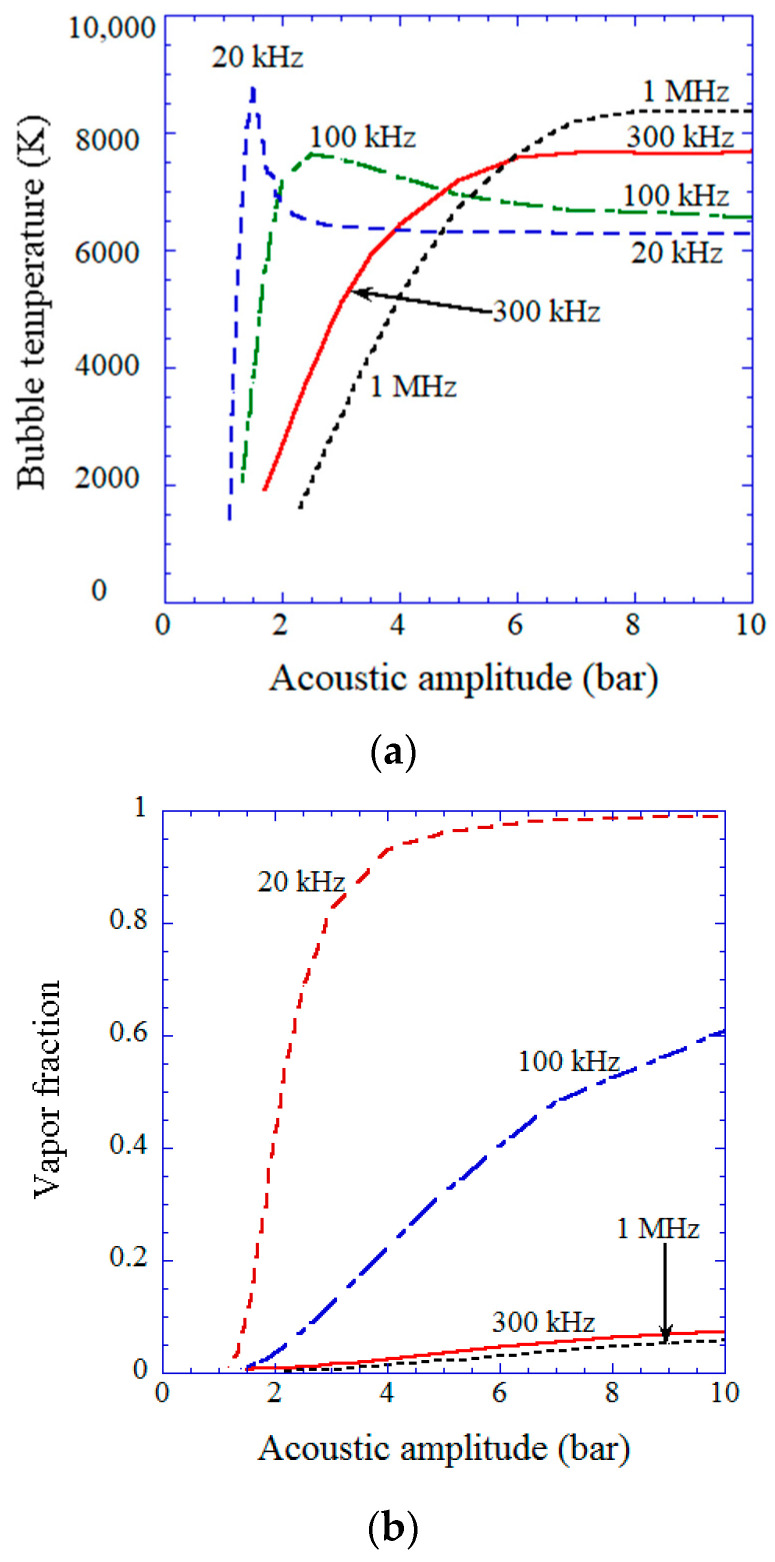
The results of the numerical simulations as a function of acoustic pressure amplitude for various ultrasonic frequencies for the first collapse of an isolated air bubble. (**a**) The temperature inside a bubble at the violent collapse. (**b**) The molar fraction of water vapor inside a bubble at the end of the violent collapse. Reprinted with permission from Ref. [53]. Copyright 2007, AIP Publishing.

Next, the dependence of amounts of chemical species produced from a cavitation bubble on ultrasonic frequency is discussed based on numerical simulations of chemical reactions inside an air bubble [53]. For relatively low ultrasonic frequencies (20 and 100 kHz), there is a peak in bubble temperature at the violent bubble collapse as a function of the pressure amplitude of ultrasound (acoustic amplitude), as shown in Figure 11a [53]. The reason is that for relatively low ultrasonic frequencies, the bubble content becomes mostly water vapor at the end of the violent bubble collapse at a relatively high acoustic amplitude because a bubble dramatically expands during the rarefaction phase of ultrasound and intense evaporation of water vapor occurs during the bubble expansion. When the bubble content is mostly water vapor, which is called a vaporous bubble, the bubble temperature at the violent collapse does not increase much because the endothermic dissociation of water vapor cools the bubble considerably [19,113,158,159]. Although the bubble collapse becomes more violent as the acoustic amplitude increases, the bubble temperature decreases for relatively high acoustic amplitudes due to the increase in the amount of water vapor trapped inside a collapsing bubble, as shown in Figure 11b [53]. This is the reason for the appearance of the peak in bubble temperature as a function of acoustic amplitude at relatively low ultrasonic frequencies. For relatively high ultrasonic frequencies (300 kHz and 1 MHz), on the other hand, the amount of water vapor trapped inside a bubble at the end of the bubble collapse is much smaller than that at relatively low ultrasonic frequencies, and thus the bubble temperature increases as the acoustic amplitude increases until it reaches a plateau as shown in Figure 11a,b [53].

**Figure 12:**
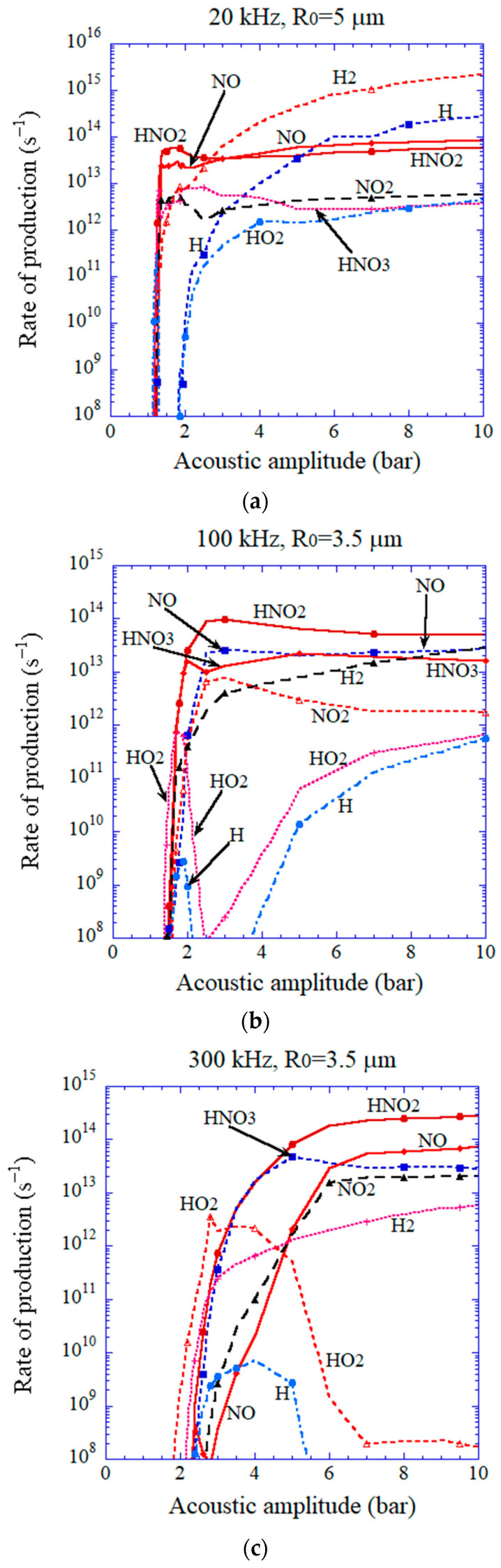

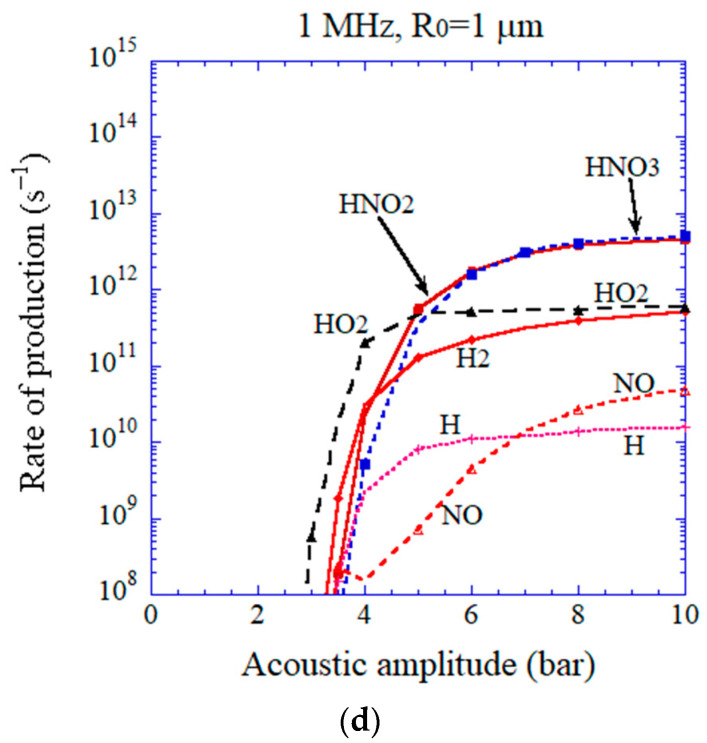
The results of the numerical simulations on the rate of production of chemical species other than oxidants inside an isolated air bubble as a function of acoustic pressure amplitude. (**a**) 20 kHz, (**b**) 100 kHz, (**c**) 300 kHz, (**d**) 1 MHz. Reprinted with permission from Ref. [53]. Copyright 2007, AIP Publishing.

When the bubble temperature is higher than about 7000 K, oxidants such as $OH,O,H_{2}O_{2},$ and $O_{3}$ are strongly consumed inside an air bubble by oxidizing nitrogen [2,53,111,116]. In addition, H atoms are also strongly consumed inside an air bubble at such a high temperature by the following chemical reactions; $H+O_{2}\to O+OH,H+O_{2}+M\to HO_{2}+M$, $H+OH\to H_{2}+O$, $H+HNO_{2}\to H_{2}+NO_{2}$, and $H+NO_{2}\to NO+OH$ [53]. As a result, the main chemical products at such a high temperature are $HNO_{2},NO,HNO_{3}$, $H_{2},$ and $NO_{2}$, as seen in Figure 12 [53].

In an ultrasonic bath, a standing wave of ultrasound is formed because ultrasound is strongly reflected by the liquid surface [2,160]. In a standing wave field, bubbles are repelled from high-acoustic-amplitude regions due to the acoustic radiation force called the primary Bjerknes force [2,121]. For example, at 20 kHz, bubbles are repelled from regions with a higher acoustic amplitude than about 1.75 bar [121,161]. In regions with a smaller acoustic amplitude than about 1.75 bar, bubbles are attracted toward the higher-acoustic-amplitude regions. Accordingly, many bubbles gather around the region with an acoustic amplitude of about 1.75 bar. Indeed, the structure of bubbles has been experimentally observed, which is sometimes called the jellyfish structure [162,163]. At about 1.75 bar at 20 kHz, the bubble temperature is higher than about 7000 K, according to Figure 11 [53]. It means that the amount of H atoms produced in an ultrasonic bath at 20 kHz is extremely small, as seen in Figure 12a [53]. It may be the reason why the rate of sonochemical reduction of $AuCl_{4}^{-}$ at 28 kHz is much lower than those at 200 and 950 kHz (Figure 1), according to Sakai et al. [69]. On the other hand, if an ultrasonic horn is used at 20 kHz, it is predicted that the amount of H atoms produced from an air bubble is much larger because the acoustic amplitude is much higher, according to Figure 12a [53,159]. However, it should be noted that under an ultrasonic horn, the bubble-bubble interaction, which is the influence of acoustic emissions of surrounding bubbles on bubble pulsation, is very strong and that the effect of the bubble-bubble interaction should be taken into account in the numerical simulations of chemical reactions inside cavitation bubbles [164,165]. Further studies are required on this topic.

Finally, the challenges faced in this field are summarized, and the outlook for the research direction is discussed. The roles of $H_{2},NO_{2}^{-},HNO_{2},H_{2}O_{2},O_{2}^{-},HO_{2}$, and hydrated electrons ($e^{-}\left(aq\right)$) in the sonochemical reduction of solutes without any additives need to be studied in more detail. The mechanism of sonochemical reduction of carbon dioxide experimentally reported recently [166] needs to be studied in more detail. Based on the detailed mechanism, conditions to enhance the sonochemical reduction of solutes need to be clarified, such as ultrasonic frequency, acoustic amplitude, type of sonochemical reactor (bath or horn), pH, etc. It will hopefully lead to industrial applications of sonochemical reduction as well as oxidation because it is a green process [167,168,169]. The same is true for chemical reactions induced by hydrodynamic cavitation (sometimes called fine-bubble technology) [170,171,172,173,174].

## 4. Conclusions

It is experimentally reported that reduction of $AuCl_{4}^{-}$ occurs in aqueous solutions without any additives such as organic materials irradiated with strong ultrasound, although oxidation reactions occur in most sonochemical reactions. Considering the high reduction potential of H atoms, the main reducing agent produced from cavitation bubbles under ultrasound in water without any additives is a H atom, according to the numerical simulations of chemical reactions inside an air or argon bubble under ultrasound. In strongly alkaline solutions, $H_{2}$ produced from cavitation bubbles could also work as a reducing agent. Hydrated electrons ($e^{-}\left(aq\right)$), which could be formed from H atoms in strongly alkaline solutions, may also work as a reducing agent. Superoxide anion ($O_{2}^{-}$), which is formed from $HO_{2}$ produced from cavitation bubbles in aqueous solutions, could possibly work as a reducing agent. Further studies are required on the role of $HNO_{2}$ in the sonochemical reduction of some solutes. The outlook for research direction is also discussed.

## Figures and Tables

**Table 1:** The amounts of chemical products that dissolve into the liquid water from the interior of a SBSL bubble (mostly an argon bubble) in one acoustic cycle according to the numerical simulation. The liquid volume in the experiment [124] was about 15 mL. Reprinted with permission from Ref. [24]. Copyright 2005, AIP Publishing.

Chemical Species	Number of Molecules per Acoustic Cycle
$H_{2}$	$3.1\times 10^{7}$
O	$1.3\times 10^{7}$
$H_{2}O_{2}$	$6.3\times 10^{6}$
H	$4.1\times 10^{6}$
${\mathrm{HNO}}_{2}$	$2.3\times 10^{6}$
${\mathrm{HO}}_{2}$	$1.1\times 10^{6}$
${\mathrm{HNO}}_{3}$	$8.4\times 10^{5}$
OH	$6.6\times 10^{5}$
NO	$2.5\times 10^{5}$
HNO	$9.5\times 10^{4}$
${\mathrm{NO}}_{2}$	$4.4\times 10^{4}$
$O_{3}$	$3.4\times 10^{4}$
N	$2.9\times 10^{4}$
${\mathrm{NO}}_{3}$	$3.1\times 10^{3}$
$N_{2}O$	$3.1\times 10^{2}$

**Table 2:** The reduction potentials of reducing agents [128,129,130].

Reducing Agent	Reaction	Reduction Potential (V)
$e^{-}\left(\mathrm{aq}\right)$	$e^{-}\left(\mathrm{aq}\right)\to e^{-}$	2.88 [128]
$H\left(\mathrm{aq}\right)$	$H\left(\mathrm{aq}\right)\to H^{+}+e^{-}$	2.31 [128]
$H_{2}$	$H_{2}+2{\mathrm{OH}}^{-}\to 2H_{2}O+2e^{-}$	0.83 [129] ^1^
${\mathrm{CH}}_{2}\mathrm{OH}$	${\mathrm{CH}}_{2}\mathrm{OH}\to \mathrm{CHOH}+H^{+}+e^{-}$	0.73 [130]
${\mathrm{CH}}_{3}\mathrm{CHOH}$	${\mathrm{CH}}_{3}\mathrm{CHOH}\to {\mathrm{CH}}_{3}\mathrm{COH}+H^{+}+e^{-}$	0.69 [130]
$O_{2}^{-}$	$O_{2}^{-}\to O_{2}\left(\mathrm{aq}\right)+e^{-}$	0.18 [128]
CO	$\mathrm{CO}+H_{2}O\to {\mathrm{CO}}_{2}+2H^{+}+2e^{-}$	0.11 [129]
${\mathrm{HO}}_{2}$	${\mathrm{HO}}_{2}\to O_{2}+H^{+}+e^{-}$	0.05 [129]
$H_{2}$	$H_{2}\to 2H^{+}+2e^{-}$	0.00 [129]
${\mathrm{NO}}_{2}^{-}$	${\mathrm{NO}}_{2}^{-}+2{\mathrm{OH}}^{-}\to {\mathrm{NO}}_{3}^{-}+H_{2}O+2e^{-}$	−0.01 [129] ^1^
NO	$\mathrm{NO}+2{\mathrm{OH}}^{-}\to {\mathrm{NO}}_{2}^{-}+H_{2}O+e^{-}$	−0.46 [129] ^1^
${\mathrm{HNO}}_{2}$	$2{\mathrm{HNO}}_{2}\to \mathrm{NO}+{\mathrm{NO}}_{3}^{-}+2H^{+}+e^{-}$	−0.52 [129]
$I^{-}$	$3I^{-}\to I_{3}^{-}+2e^{-}$	−0.54 [129]
$H_{2}O_{2}$	$H_{2}O_{2}\to O_{2}+2H^{+}+2e^{-}$	−0.70 [129]
NO	$\mathrm{NO}+H_{2}O\to {\mathrm{NO}}_{2}+2H^{+}+2e^{-}$	−1.05 [129]

^1^ $\left[{\mathrm{OH}}^{-}\right]\approx 1$ mol L^−1^.

**Table 3:** The amounts of chemical products that dissolve into the liquid water from the interior of an initial air bubble in one acoustic cycle according to the numerical simulation. Reprinted with permission from Ref. [24]. Copyright 2005, AIP Publishing.

Chemical Species	Number of Molecules per Acoustic Cycle
${\mathrm{HNO}}_{2}$	$4.0\times 10^{7}$
${\mathrm{HNO}}_{3}$	$3.7\times 10^{7}$
O	$1.6\times 10^{7}$
$H_{2}O_{2}$	$5.1\times 10^{6}$
$O_{3}$	$2.7\times 10^{6}$
${\mathrm{HO}}_{2}$	$2.3\times 10^{6}$
${\mathrm{NO}}_{3}$	$1.1\times 10^{6}$
$H_{2}$	$1.0\times 10^{6}$
OH	$9.9\times 10^{5}$
${\mathrm{NO}}_{2}$	$3.9\times 10^{5}$
$N_{2}O$	$3.0\times 10^{5}$
NO	$1.3\times 10^{5}$
H	$1.1\times 10^{5}$
HNO	$2.8\times 10^{4}$
N	$2.7\times 10^{3}$
$N_{2}O_{5}$	$6.8\times 10^{2}$

**Table 4:** The oxidation potentials of oxidants [129,146].

Oxidant	Reaction	Oxidation Potential (V)
OH	$\mathrm{OH}+H^{+}+e^{-}\to H_{2}O$	2.81 [126]
O	$O+2H^{+}+2e^{-}\to H_{2}O$	2.42 [126]
$O_{3}$	$O_{3}+2H^{+}+2e^{-}\to O_{2}+H_{2}O$	2.07 [126]
$H_{2}O_{2}$	$H_{2}O_{2}+2H^{+}+2e^{-}\to 2H_{2}O$	1.78 [126]
${\mathrm{NO}}_{2}^{-}$	$2{\mathrm{NO}}_{2}^{-}+6H^{+}+4e^{-}\to N_{2}O+3H_{2}O$	1.40 [116]
${\mathrm{HNO}}_{2}$	$2{\mathrm{HNO}}_{2}+4H^{+}+4e^{-}\to N_{2}O+H_{2}O$	1.30 [116]
${\mathrm{NO}}_{2}^{-}$	${\mathrm{NO}}_{2}^{-}+2H^{+}+e^{-}\to \mathrm{NO}+H_{2}O$	1.20 [116]
${\mathrm{HNO}}_{2}$	${\mathrm{HNO}}_{2}+H^{+}+e^{-}\to \mathrm{NO}+H_{2}O$	1.00 [116]
${\mathrm{AuCl}}_{4}^{-}$	${\mathrm{AuCl}}_{4}^{-}+3e^{-}\to \mathrm{Au}+4{\mathrm{Cl}}^{-}$	1.00 [116]

## Data Availability

Not applicable.
